# Draft Genome Sequence of *Porphyromonas gingivalis* Strain Ando Expressing a 53-Kilodalton-Type Fimbrilin Variant of Mfa1 Fimbriae

**DOI:** 10.1128/genomeA.01292-15

**Published:** 2015-11-05

**Authors:** Takatsugu Goto, Keiji Nagano, Hideki Hirakawa, Kaori Tanaka, Fuminobu Yoshimura

**Affiliations:** a Division of Anaerobe Research, Life Science Research Center, Gifu University, Gifu, Japan; b Department of Microbiology, School of Dentistry, Aichi Gakuin University, Aichi, Japan; c Department of Technology Development, Kazusa DNA Research Institute, Chiba, Japan; d United Graduate School of Drug Discovery and Medical Information Sciences, Gifu University, Gifu, Japan

## Abstract

Periodontopathic *Porphyromonas gingivalis* strain Ando abundantly expresses a 53-kDa-type Mfa1 fimbria. Here, we report the draft genome sequence of Ando, with a size of 2,229,994 bp, average G+C content of 48.4%, and 1,755 predicted protein-coding sequences.

## GENOME ANNOUNCEMENT

*Porphyromonas gingivalis*, a Gram-negative anaerobe, is a major contributor to periodontal diseases (1, 2). *P. gingivalis* expresses two types of fimbriae, FimA and Mfa1 (3), and it is known that there are six genotypes in genes encoding the major fimbrilin of FimA fimbriae (4). Recently, we reported that there were variants even in the major fimbrilin of Mfa1 fimbriae, the Mfa1 (75-kDa) and 53-kDa types (5). The published complete genome sequences of *P. gingivalis* strains, including W83 (6), ATCC 33277 (7), TDC60 (8), and HG66 (9), show that they all possess a DNA sequence corresponding to the gene encoding a 75-kDa fimbrilin. Here, we report the draft genome sequence of *P. gingivalis* strain Ando, which abundantly expresses a 53-kDa-type Mfa1 fimbria.

The genomic DNA of Ando was sequenced using the Illumina HiSeq 2000 (90- or 100-bp paired-end reads, with an average 186-bp insert size). After the raw sequences were trimmed and their quality filtered (Sanger QV, ≥10), the remaining 5,247,742 reads, with approximately 224-fold genome coverage, were assembled *de novo* using Velvet 1.2.08 (the best *k*-mer, 91 bp). The final draft assembly consists of 112 contigs (>180 bp), for a total length of 2,229,994 bp (*N*_50_, 55,724 bp) and a G+C content of 48.4%. Protein-coding sequences (CDSs), tRNA genes, and clustered regularly interspaced short palindromic repeats (CRISPRs) were predicted by MetaGeneAnnotator (10), tRNAscan-SE 1.23 (11), and CRISPRFinder (12), respectively. Functional annotation of CDSs comes from BLASTP searches against NCBI’s nonredundant (NR) protein database (ftp://ftp.ncbi.nlm.nih.gov/blast/db/FASTA/nr.gz).

A total of 1,755 CDSs, 47 tRNA genes, 4 rRNA genes, and 3 CRISPRs were predicted in the Ando genome. PHAST (13) did not detect any prophage region. A comparative analysis of the CDSs among Ando and the other four *P. gingivalis* strains using CD-HIT 4.6.4 (14) (cutoff, 90% sequence similarity, 90 to 110% length coverage) showed significant similarities, with 87.7% similarity to TDC60, 86.2% similarity to HG66, 85.1% similarity to W83, and 85.1% similarity to ATCC 33277. A reciprocal best hit analysis of Ando chromosomal CDSs against W83 chromosomal CDSs (6) using BLASTP (*E* value cutoff, 10^-10^) predicted potential virulence genes in Ando (described as numbers in Ando out of numbers in W83): 1 of 1 hemolysin, 9 of 13 adhesin (e.g., hemagglutinin), 62 of 67 evasion proteins (e.g., glycosyltransferase), 10 of 10 invasion proteins, 16 of 16 stress response proteins, 5 of 5 antibiotic resistance proteins, and 40 of 41 peptidases. The gene encoding the 53-kDa fimbrilin (PGANDO_1061) in Ando was not found in the other four *P. gingivalis* strains. However, the PGANDO_1061 gene was located at the same locus of the *mfa1* gene in other strains. We found several other genes that were specifically detected in Ando (e.g., plasmid-related proteins, CRISPR-associated proteins, and virulence-associated protein E). Further comparative genomic and functional analyses with other strains that express 53-kDa fimbrilins will help understand the pathogenic mechanism of *P. gingivalis*.

### Nucleotide sequence accession numbers.

This genome sequence and raw sequence reads have been deposited, respectively, in DDBJ/ENA/GenBank and DDBJ Sequence Read Archive under the accession numbers BCBV01000001 to BCBV01000112 and DRA003978 (BioProject PRJDB4201).
